# Plexcitonic Quantum Light Emission from Nanoparticle-on-Mirror Cavities

**DOI:** 10.1021/acs.nanolett.1c04872

**Published:** 2022-03-14

**Authors:** Rocío Sáez-Blázquez, Álvaro Cuartero-González, Johannes Feist, Francisco J. García-Vidal, Antonio I. Fernández-Domínguez

**Affiliations:** † Departamento de Física Teórica de la Materia Condensada and Condensed Matter Physics Center (IFIMAC), Universidad Autónoma de Madrid, 28049 Madrid, Spain; ‡ Vienna Center for Quantum Science and Technology, Atominstitut, TU Wien, 1040 Vienna, Austria; ¶ Mechanical Engineering Department, ICAI, Universidad Pontificia Comillas, 28015 Madrid, Spain; § Institute of High Performance Computing, Agency for Science, Technology, and Research (A*STAR), Singapore 138632, Singapore

**Keywords:** plexciton, nanocavity, quantum emitter, antibunching, quantum light

## Abstract

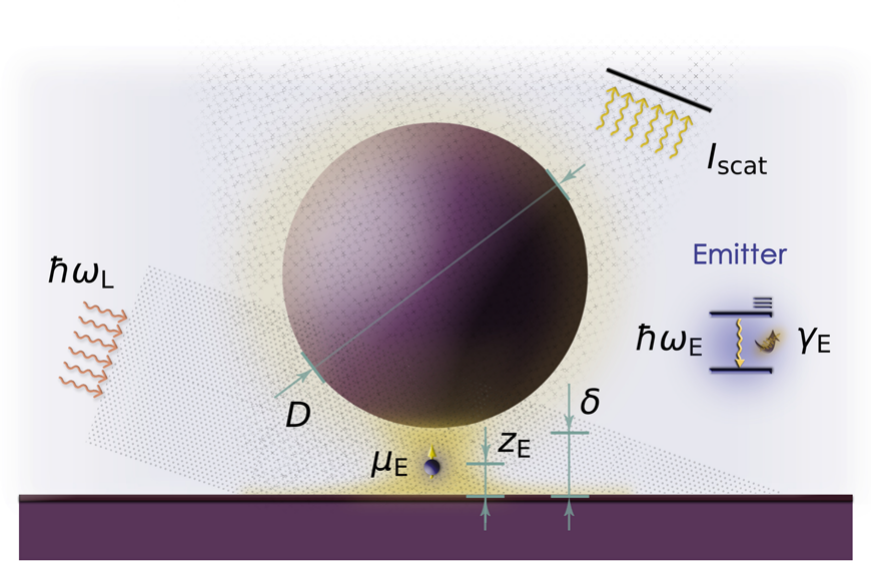

We investigate the quantum-optical properties of the light emitted by a nanoparticle-on-mirror cavity filled with a single quantum emitter. Inspired by recent experiments, we model a dark-field setup and explore the photon statistics of the scattered light under grazing laser illumination. Exploiting analytical solutions to Maxwell’s equations, we quantize the nanophotonic cavity fields and describe the formation of plasmon–exciton polaritons (or plexcitons) in the system. This way, we reveal that the rich plasmonic spectrum of the nanocavity offers unexplored mechanisms for nonclassical light generation that are more efficient than the resonant interaction between the emitter natural transition and the brightest optical mode. Specifically, we find three different sample configurations in which strongly antibunched light is produced. Finally, we illustrate the power of our approach by showing that the introduction of a second emitter in the platform can enhance photon correlations further.

Surface plasmons (SPs) have been largely exploited to tailor the classical (spatial and temporal) characteristics of the electromagnetic (EM) fields produced by single molecules and quantum dots.^1,2^ Two paradigmatic examples of such manipulation are the reshaping of their dipolar radiation pattern by directional nanoantennas^3,4^ or the Purcell reduction of their natural lifetime in nanogaps.^5−7^ In recent years, this ability of SPs for EM control has also been transferred into the quantum arena.^8,9^ Initial efforts focused on the imprinting of nonclassical features, such as entanglement,^10^ quadrature squeezing,^11^ or sub-Poissonian statistics,^12^ in plasmonic waves through the incident, driving fields. In this context, quantum emitters (QEs) were used as the optical sources that allowed the near-field launching of confined single plasmons in metallic nanowires.^13,14^

The quest for plasmon-assisted generation of radiative quantum states of light, propagating in free-space and into the far-field, has attracted much attention lately.^15^ Devices based on guiding geometries decorated with in- and out-coupling elements have been thoroughly investigated.^16,17^ SPs suffer heavily from metallic absorption in these extended systems. For this reason, nanocavities have emerged as an alternative for nonclassical light sources of smaller dimensions. Importantly, these nanostructures also make it possible to fully harness the large density of photonic states associated with SPs.^18^ Theoretical studies have shown that the weak interaction between a single QE and a metallic nanosphere gives rise to moderate photon antibunching and reduction of quantum-optical fluctuations.^19−22^ Accordingly, the measurement of second-order correlation functions below unity is taken as proof of the single emitter operation in experiments on the Purcell effect in plasmonic antennas.^23,24^

The realization of stronger quantum nonlinearities with larger near-to-far-field transfer efficiencies requires nanocavity–QE samples that function in the strong-coupling regime.^25^ This leads to the formation of hybrid SP–QE states, usually termed plexcitons,^26−28^ whose properties can be tuned through the admixture of the interactive character of excitons and the coherence and ubiquity of photons. This phenomenon not only offers new avenues for light generation but also lies at the core of the emergent field of polaritonic chemistry.^29,30^ The accurate description of the signature of SP–QE interactions in far-field optical signals relies on the EM quantization in nanometer-sized, lossy structures, which is an area of intense activity at the moment.^31−34^ Concurrently, plexciton formation in various QE–nanocavity platforms has been realized experimentally,^35−40^ and a number of theoretical models have investigated the emergence of photon correlations in these systems.^41−44^

In this Letter, we theoretically investigate the quantum-optical properties that plexciton strong coupling induces in the light scattered by a plasmonic cavity^45,46^ in a dark-field setup.^47,48^ Through radiative-corrected quasi-static EM calculations,^49,50^ we describe the near-field and radiative characteristics of the SP modes sustained by the structure as well as their interaction with a molecule placed at its gap. We first characterize the response of the bare cavity under grazing laser excitation. Second, we describe the far-field intensity and second-order correlation spectra for the most experimentally explored configuration:^35,36,38^ QE at resonance with the brightest, dipolar (lowest in frequency) SP mode. We next perform a comprehensive study of the dependence of photon correlations on the detuning between QE and laser frequencies as well as on the cavity gap size. Thus, we reveal three different parameter ranges in which strong antibunching can be attained. Finally, we illustrate the power of our approach by introducing a second QE in the system. We find that the second-order correlation function can be further reduced this way,^51^ thanks to the emergence of new pathways for destructive quantum interference in the plexciton ladder.

## Theoretical Modeling

**Figure 1:**
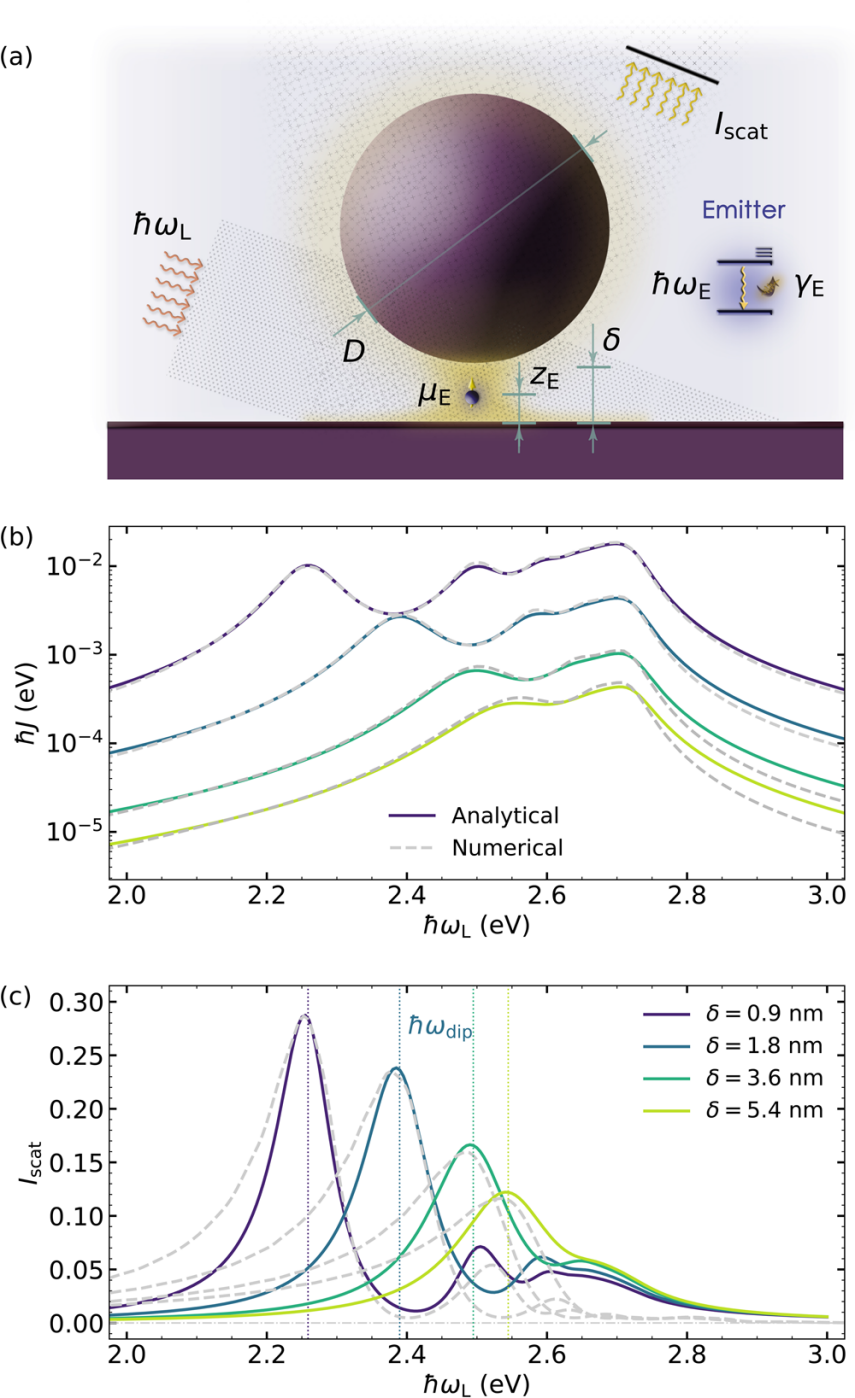
(a) Sketch of the system, composed of a single QE coupled to the SP fields within the gap of a NPoM cavity in a dark-field-like setup. The inset shows the two-level scheme modeling the emitter. (b) Spectral density *J*(ω) at the gap center for different values of the gap size δ (with *D* = 30 nm). (c) Normalized dark-field scattering spectra for the bare cavities above. Solid lines represent *I*_SP_ given by eq 4, while dashed lines correspond to scattered intensity obtained by numerical EM calculations. Vertical dotted lines indicate the position of the lowest-order (dipolar) plasmon mode with energy ℏω_dip_ for each gap size.

Figure 1a is a sketch of the system of interest: an archetypal nanoparticle-on-mirror (NPoM) cavity^52,53^ formed by a 30 nm diameter nanosphere separated by a few-nanometer gap from a planar substrate.^35,46^ Both are metallic with permittivity given by a Drude fitting for silver, ϵ(ω) = ϵ_∞_ – ω_p_^2^/ω(ω + *i*γ_m_), where ω_p_ = 8.91 eV, ϵ_∞_ = 9.7, and γ_m_ = 0.06 eV. For simplicity, the background refractive index is set to unity. We employ an analytical, two-dimensional model that we recently developed (see ref (50) for more details) to describe the SP modes sustained by this geometry (fully defined by the diameter, *D*, and gap size, δ). This tool is based on quasi-static solutions to Maxwell’s equations and is refined by means of the so-called radiative-reaction correction,^54,55^ yielding an excellent agreement with numerical EM simulations. A QE is placed in the NPoM gap. It is characterized by its transition dipole moment, μ_E_, transition frequency, ω_E_, and radiative, γ_rad_, and nonradiative, γ_nrad_, decay rates as well as its dephasing rate, γ_deph_ (which we keep small enough to ensure that dephasing-induced spectral asymmetries are negligible in our plexcitonic systems^56,57^). Note that the QE radiative decay rate is simply γ_rad_ = ω_E_^3^μ_E_^2^/(3πϵ_0_ℏ*c*^3^)^58^ (where ϵ_0_ is the vacuum permittivity and *c* is the speed of light) and its nonradiative decay is set by its intrinsic quantum yield QY = γ_rad_/(γ_rad_ + γ_nrad_). The hybrid NPoM–QE sample is driven by a grazing laser field of frequency ω_L_ and amplitude *E*_L_, mimicking a dark-field-like illumination.

Our quasi-static treatment allows the labeling of the NPoM modes in terms of two quantum numbers, their azimuthal order, *n* = 1, 2, 3, ..., and the odd/even parity of their associated EM fields across the gap center, σ = ±1.^55^ Here, to simplify the notation, we combine both in a single index α = {*n*, σ}. Our theory also yields their natural frequencies, ω_α_, and broadenings, γ_α_ = γ_m_ + γ_α_^rad^. Note that plasmonic absorption is the same for all SPs, given by the Drude damping, and their radiative decay is proportional to the square of their dipole moment, γ_α_^rad^ ∝ μ_α_^2^ (where the usual free-space expression has to be corrected by the image charge distribution induced in the metal substrate^50^). We also obtained closed expressions for the QE–SP coupling strengths, *g*_α_, and their dependence on the QE parameters (position and natural frequency).

In the rotating frame,^59^ set by the laser frequency, and under the rotating-wave approximation,^60^ the Hamiltonian for the setup in Figure 1a is^51^
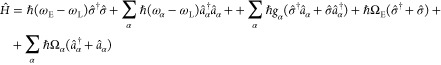
1where â_α_ and σ̂ are the SP and QE annihilation operators, respectively. Note that the third term in eq 1 describes the light–matter coupling, where ℏ*g*_α_ = **E**_α_^(1)^ · **μ**_E_ and **E**_α_^(1)^ is the quantized one-photon field strength of mode α at the QE position. Then, ℏΩ_E_ = **E**_L_ · **μ**_E_ and ℏΩ_α_ = **E**_L_ × **μ**_α_; both are the coherent pumping amplitudes. Note that dipole moments and incident fields are oriented vertically in Figure 1a. The master equation for the steady state of the system including SP damping and QE decay and dephasing is
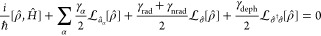
2where the Lindblad terms have the usual form .

## Results and Discussion

Before investigating far-field optical signatures of light–matter interactions in the hybrid QE–SP system, we employ our theory to characterize the bare plasmonic cavity first. We compute the spectral density by weighting the local density of photonic states and the QE–SP coupling strength at the NPoM gap. We have recently shown that this is given by^34^
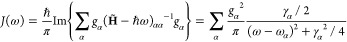
3where  is equal to the coefficient matrix of the SP modes in the effective non-Hermitian Hamiltonian governing the coherent evolution in the Lindblad master equation.

Figure 1b renders the spectral density at the center of the NPoM gap, *z*_E_ = 0.5 δ (*z* = 0 corresponds to the substrate surface), for cavities with *D* = 30 nm and δ ranging from 0.9 (purple) to 5.4 nm (light green). The coupling constants are proportional to the QE dipole moment, . They were evaluated at μ_E_ = 0.55 e·nm, the value that we consider in our plexcitonic systems. As previously reported,^28^ the smaller the gap, the larger is *J*(ω). In all cases, the spectra present a low-frequency maximum originating from the brightest, dipolar SP mode, α = {1, 1}, which redshifts with decreasing δ, and another maximum in the vicinity of the asymptotic surface plasmon frequency  due to the pseudomode that results from the spectral overlapping of high order SPs.^61^ For small enough gap sizes, the contributions from quadrupolar and higher order, even modes (specifically, α = {1 < *n* < 5, 1}) are also apparent. Gray dashed lines correspond to quasi-static numerical simulations performed with COMSOL Multiphysics, showing an excellent agreement with our analytical predictions. More details are provided in the Supporting Information, in which Figure S1 proves the moderate impact of nonlocal effects in the metal permittivity, even for δ = 0.9 nm, by means of calculations based on a hydrodynamical Drude model.^62,63^Figure S2 presents the comparison against full electrodynamic simulations and reveals the small effect of retardation in *J*(ω) for *D* = 30 nm. It also plots far-field radiation patterns for the lowest SP modes, which due to retardation and despite their different near-field multipolar character, present a similar dipolar-like character. Moreover, inspired by recent experiments,^18,39^ in Figure S3, we explore the impact of geometric flat facets at the gap cavity, and Figure S4 gives a full characterization of the SP modes (through their dipole moments, μ_α_, and natural frequencies, ω_α_) for NPoM cavities with different δ values.

We focus next on the far-field response of the bare NPoM structure. We compute the scattering spectrum by solving eq 2 by removing all the QE-related terms. Once the SP steady-state density matrix, ρ̂_SP_, is known, the far-field scattering intensity can be computed as
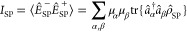
4where *Ê*_SP_^–^ = Σ_α_μ_α_*â*_α_^†^ is the (negative frequency part of the) electric far-field operator. For simplicity, we are dropping the Dyadic Green’s function in the definition of the electric field operator, which would account for the spatial pattern of the cavity fields. Importantly, the cross terms in eq 4 reflect the emergence of superposition effects in the photon emission from different SP modes.

Figure 1c plots the scattering spectra for the NPoM configurations in Figure 1b. Dashed lines correspond to numerical simulations of the scattering efficiency (defined as the cross section normalized to physical size) in the quasi-static limit, while solid lines plot the prediction from eq 4. Note that the latter have been scaled vertically (by the same factor for all δ values) to facilitate the comparison between both sets of data. We can observe that the spectra are governed by a large peak at laser frequencies in the vicinity of the lowest, dipolar SP (α = {1, 1}). The condition ω_L_ = ω_dip_ is indicated by vertical dotted lines in all cases. Higher order SP maxima are also evident, specially at small δ values. On the contrary, there is not any pseudomode signature in the scattering signal, as expected from the dark character of the SP modes that form it. The NPoM spectra in Figure 1c present scattering minima at laser frequencies between SP resonances. These are the so-called invisibility dips that emerge (more clearly in log scale) due to the destructive interference in the photon emission by different plasmonic channels.^55^

Next, we place a vertically oriented QE at the center of the gap of the NPoM geometries in Figure 1. Reproducing previous experimental setups, we set the QE frequency at resonance with the dipolar SP, ω_E_ = ω_dip_, which is different for each δ value. This way, the signature of the QE–SP interaction is expected to be most apparent in the far-field. We take QY = 0.65, in agreement with values reported for molecular dyes, such as Atto 647N.^39^ The associated radiative and nonradiative decay rates are therefore in the 10^–6^–10^–7^ eV range (note that these depend on ω_E_). Additionally, we consider a QE dephasing rate of γ_deph_ = 1 meV.^64^ The spectra for the hybrid NPoM–QE system can be obtained from the steady-state density matrix solution, ρ̂, for the full master equation in eq 2,
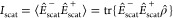
5where in order to account for the open character of the plasmonic cavity, the electric field operator, *Ê*_scat_^–^ = *Ê*_SP_^–^ + μ_E_σ̂^†^, now includes the emission from the molecule itself.^51^ Note that we can also compute the density matrix, ρ̂_E_, and scattering intensity for the free-standing emitter, obtained from the QE terms in eq 2 and *I*_E_ = μ_E_^2^tr{σ̂^†^σ̂ρ̂_E_}.

**Figure 2:**
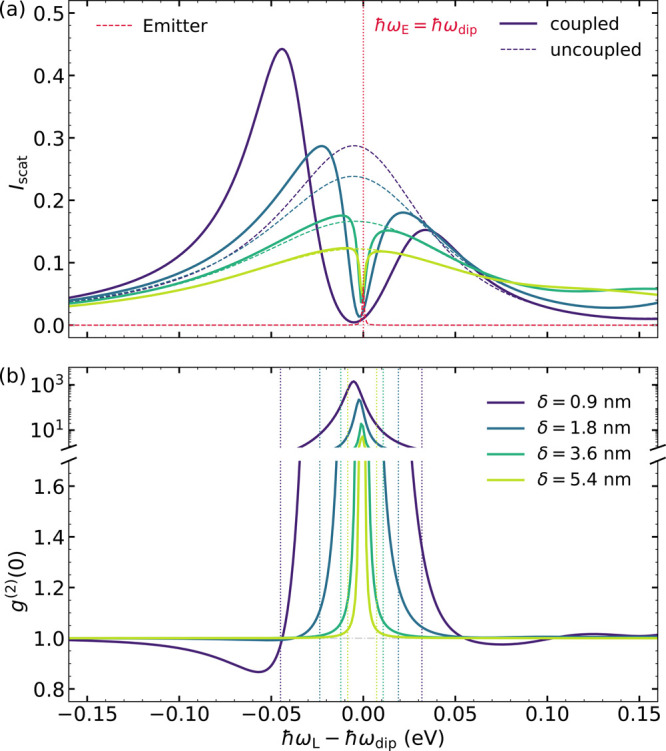
(a) Far-field spectra for the bare cavities in Figure 1 (dashed lines) and the hybrid SP–QE samples (solid lines) that result from introducing a vertically oriented molecule at the gap center. *I*_scat_ is plotted against the detuning between the incident laser and the dipolar SP, ℏω_L_ – ℏω_dip_, and only in the vicinity of this cavity mode. The dashed red line represents the free-standing QE spectrum, which is, in all cases, at resonance with the dipolar SP, ω_E_ = ω_dip_ (vertical dotted line). (b) Zero-delay second-order correlation function, *g*^(2)^(0), versus laser detuning for the same NPoM–QE configurations in (a). Vertical dotted lines indicate the plexciton frequencies in the one-excitation manifold for each gap size.

Figure 2a shows the scattering intensity versus ω_L_ – ω_dip_, the laser detuning with respect to the dipolar SP, which allows the direct comparison between different cavities. Dashed lines correspond to the Lorentzian-like spectral profile of *I*_SP_ for all structures, while solid lines plot the spectra for the plexcitonic samples. For reference, the red dotted line shows *I*_E_ normalized to the nanoparticle size, whose line width is given by γ_E_ = γ_rad_ + γ_nrad_ + γ_deph_ ≃ 1 meV. For large gaps and therefore lower QE–SP coupling strengths, the presence of the molecule leads to the appearance of a scattering dip at ω_L_ = ω_E_ of width similar to γ_E_. This phenomenology, closely related to the electromagnetic induced transparency, is in accordance with that reported previously for single metallic nanoparticles in the weak-interaction regime.^20^ For small δ values, the far-field spectra develop a well-defined Rabi doublet line shape. This is the fingerprint of the onset of strong coupling between the bright plasmon mode and the molecule.^35^ These two scattering maxima originate from the upper and lower plexcitonic states that have been formed in the cavity (see below). Two different mechanisms contribute to make the intensity of the lower plexciton larger than the upper one. On the one hand, the former (latter) results from the constructive (destructive) interference of the SP and QE emission channels.^51^ On the other hand, it has been shown that, despite being highly detuned, higher frequency, neighboring SP modes can also increase the Rabi asymmetry in these systems.^50^

Our approach enables us to characterize the light scattered by the NPoM–QE system beyond the intensity spectra above. We can employ it to analyze the scattered photon statistics through the so-called zero-delay second-order correlation function^60^
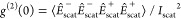
6which gives the probability of detecting two coincident photons in the far-field. Although not discussed above, the bosonic character of SPs yields *g*^(2)^(0) = 1 for the bare NPoM cavities in Figure 1. In the following, we will explore the conditions in which the plexcitonic system deviates from these Poissonian statistics with special focus on the emergence of negative correlations, antibunching, or sub-Poissonian statistics. With all these terms, we will refer to photon emission characterized by a second-order correlation function below unity, *g*^(2)^(0) < 1.

In Figure 2b, we plot the zero-delay second-order correlation function for the NPoM–QE samples in panel (a). Vertical dotted lines indicate the two plexciton frequencies in the first manifold for all geometries (which coincide with the scattering intensity maxima^41^). We can observe that *g*^(2)^(0) ≫ 1, bunched emission, or more rigorously, super-Poissonian statistics, takes place between them. The maximum in *g*^(2)^(0) occurs at ω_L_ ≃ ω_E_ and redshifts and increases with decreasing gap size (larger QE–SP coupling). Only for δ = 0.9 nm (purple line) negative photon correlations are apparent. A region of moderate antibunching, *g*^(2)^(0) > 0.8, develops for laser frequencies slightly below the lower plexciton frequency (note that an even shallower dip also occurs at ω_L_ above the upper plexciton). The correlation spectra overlap with those obtained by neglecting photon emission by SP modes different from the dipolar one, which therefore do not play any role in this particular NPoM–QE configuration.

The negative correlations observed in Figure 2b can be attributed to the so-called *photon blockade effect*,^65−67^ where the presence of an excitation in the system prevents the absorption of a second photon of the same frequency due to the anharmonicity of the plexciton ladder. This phenomenon becomes stronger as the light–matter interaction strengthens, which means that smaller gap sizes or larger QE dipole moments would be required to reduce *g*^(2)^(0) further. However, another effect yielding sub-Poissonian photon emission, known as interference-induced or *unconventional antibunching*, exists.^68,69^ Thoroughly analyzed in single-mode semiconductor microcavities,^70^ it develops only when the driving laser is far from resonance and is due to destructive quantum interference among different de-excitation pathways in the system. In the following, we investigate the emergence of both antibunching mechanisms in our plexcitonic samples, exploring the full richness of the NPoM spectrum through the emitter and laser frequencies and the emitter position.

**Figure 3:**
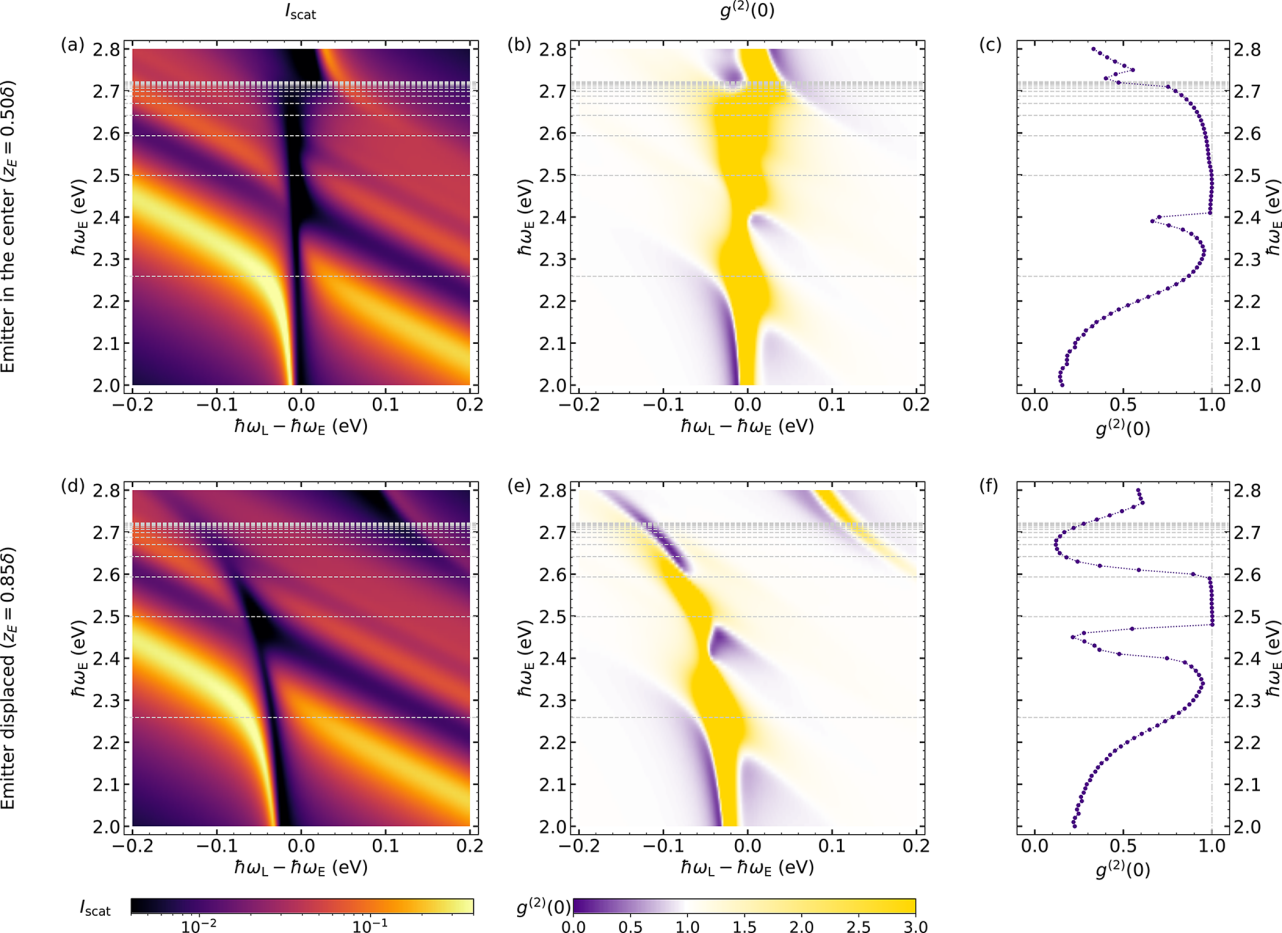
Scattering intensity *I*_scat_ (first column) and second-order correlation function *g*^(2)^(0) (second column) versus laser-QE detuning ℏω_L_ – ℏω_E_ and QE frequency, ℏω_E_ for emitter at the center of the gap (top, a–c) or vertically displaced (bottom, d–f). Panels in the third column plot the minimum of *g*^(2)^(0) as a function of the emitter frequency extracted from (b) and (e). In all panels, horizontal dotted lines indicate the position of the NPoM SP frequencies.

Figure 3 shows intensity (left panels) and second-order correlation function (central panels) maps as a function of the detuning of the laser with respect to the QE frequency (horizontal axes) and the emitter frequency itself (vertical axes). The gap size is fixed to δ = 0.9 nm, and two different emitter positions are considered: at the center of the gap, *z*_E_ = 0.5δ (top, a–c), and displaced vertically toward the nanoparticle surface, *z*_E_ = 0.85δ (bottom, d–f). In these panels, the SP frequencies, ω_α_, are marked by horizontal dotted lines. Note that the purple solid lines in Figure 2 correspond to horizontal cuts of Figure 3a,b in the vicinity of the dipolar SP. In this range of QE frequencies and below it (ω_E_ ≲ ω_dip_ = 2.26 eV), *I*_scat_ develops a clear Rabi doublet line shape, associated with the two plexcitons that emerge from the strong coupling of QE and dipolar SP. For red-detuned QEs, the lower (upper) plexciton has a more emitter-like (plasmon-like) character, and its position approaches ω_E_ (ω_dip_). On the contrary, for blue detuned QEs, the signature of higher order SPs becomes apparent, and *I*_scat_ reproduces a similar anticrossing phenomenology as that around the dipolar SP mode. The intensity maps for both emitter positions are similar with a remarkable difference: while the scattering dip between upper and lower plexcitons is always at ω_L_ = ω_E_ at the gap center (a), it redshifts with increasing QE frequency for *z*_E_ = 0.85δ (d). This is a direct consequence of the large coupling to the plasmonic pseudomode that the emitter experiences when it is placed in close proximity to the nanoparticle boundary. This is evident in the far-field spectra even for ω_E_ significantly detuned from the pseudomode frequency.^50^

The photon correlation maps in Figure 3b,d show that *g*^(2)^(0) has a higher sensitivity on the QE position than *I*_scat_. Both panels expose that bunching emission (yellow, *g*^(2)^(0) > 1) takes place at the conditions for plexciton anticrossing, where *I*_scat_ is minimum. They also reveal that much stronger negative correlations than the resonant (ω_E_ = ω_dip_) configuration considered in Figure 2 can be achieved by exploiting the full plasmonic spectrum of NPoM cavities. To clarify the degree of antibunching attainable in these systems, Figure 3c,f plots the spectral minimum of *g*^(2)^(0) as a function of the QE frequency. For both *z*_E_ values, a region of sub-Poissonian statistics is apparent at emitter frequencies below the dipolar SP, which becomes stronger and spectrally broader for lower ω_E_. As we discussed above, at laser frequencies slightly below the emitter frequency, these negative correlations are generated via the photon blockade effect, yielding *g*^(2)^(0) values below 0.2. On the contrary, a weaker interference-induced antibunching takes place in this region but for ω_L_ > ω_E_; see Figure 3b,e. The Supporting Information sheds further insights into these photon correlations by analyzing their dependence on two aspects: the number of SP modes contributing to *J*(ω), in Figures S6 and S7, and the QE nonradiative decay and dephasing rates, γ_nrad_ and γ_deph_, in Figure S5. The results therein evidence that photon blockade and interference-induced mechanisms take place in the system at laser frequencies slightly below and above ω_dip_, respectively.

Another region yielding *g*^(2)^(0) < 1 in Figure 3b,e occurs at QE frequencies approaching the pseudomode. Note that, contrary to previous studies,^28,61^ we included as many SP modes in the description of the plasmonic pseudomode as required to reach convergence in the correlation spectra calculations. We can observe that negative correlations in this window are weaker than below the dipolar SP and, as discussed below, they have a different, interference-induced, origin. These become more apparent for *z*_E_ = 0.85δ, since the coupling to the higher-energy plasmon modes increases this way.^28^ Antibunching also takes place in a third NPoM–QE configuration at QE frequencies in between the dipolar (lowest) and the quadrupolar (second lowest, α = {2, 1}) SP cavity modes (2.3 eV ≲ ω_E_ ≲ 2.5 eV), exactly at the parameter range where a scattering (invisibility) dip, due to destructive interference effects in the emission by these two SPs, evolves in *I*_scat_.^55^ We can therefore conclude that this phenomenon emerges not only in the intensity spectrum but also in the photon statistics. The QE position weights the relative coupling between the emitter and both cavity modes and, thus, the strength of the interference that suppresses two-photon processes, which seems to be larger (reaching *g*^(2)^(0) below 0.2) for displaced emitters. These findings are further clarified in the Supporting Information, which shows that the contribution of other SP modes, apart from the dipolar and quadrupolar ones, to the photon antibunching in this spectral window is not negligible; see Figure S8. This fact indicates that the full complexity of the plasmonic spectrum of NPoM cavities must be taken into account in order to assess their performance for nonclassical light generation. Moreover, calculations in Figure S5 also show that *g*^(2)^(0) decreases with increasing γ_nrad_ at ω_L_ ≈ 2.40 eV, which proves the interference-induced origin of negative correlations in this system configuration.

**Figure 4:**
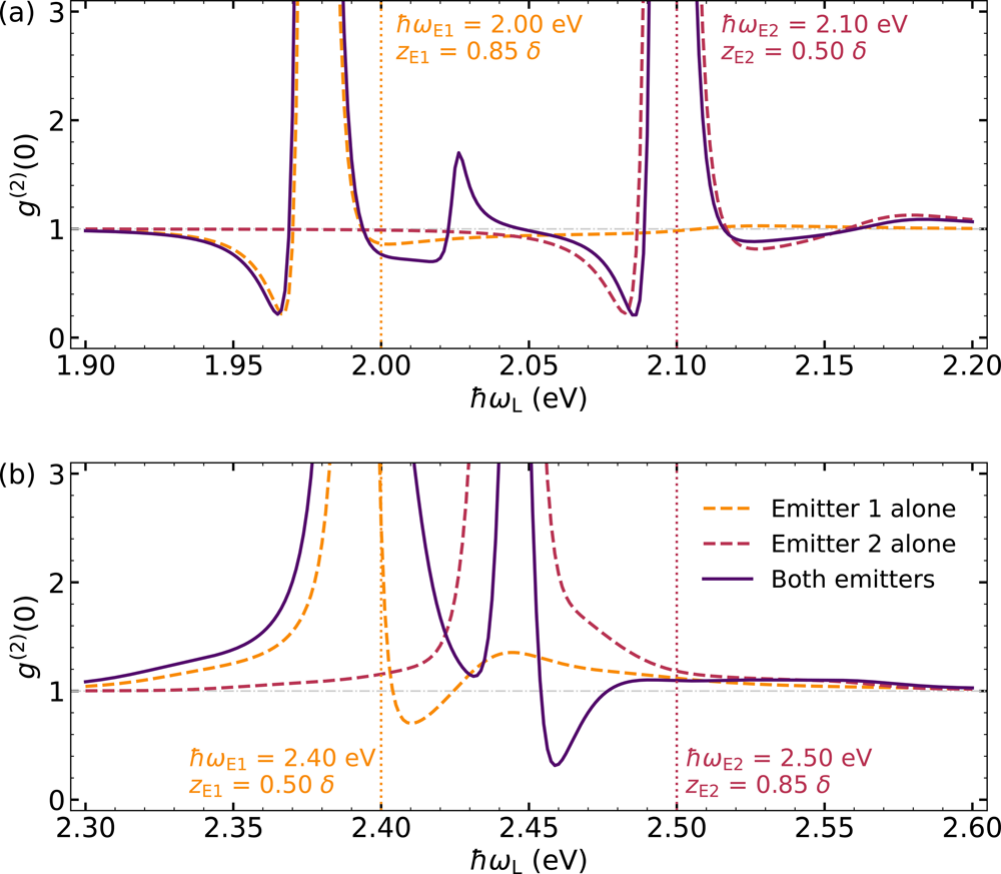
Second-order correlation function versus laser frequency, ω_L_, for two different plexcitonic systems containing two QEs (one at the gap center, another vertically displaced). The QE frequencies are either red-detuned with respect to the dipolar plasmon (a) or lying within the scattering (invisibility) dip (b). Here, *g*^(2)^(0) for the two-emitter configurations (solid lines) are compared against the corresponding single-emitter calculations (dashed lines). Vertical dotted lines indicate the values of ℏω_E_ in each case.

Lastly, we investigate whether, as previously reported for single-mode cavity models,^41^ the presence of a second emitter may be beneficial for the generation of nonclassical, antibunched light in QE–SP systems. We consider two vertically oriented emitters hosted in the small gap cavity above (δ = 0.9 nm, *D* = 30 nm). The two QE positions are chosen to be the same as in Figure 3: *z*_E1_ = 0.50δ and *z*_E2_ = 0.85δ. Figure 4 plots the second-order correlation function versus the laser frequency for two different configurations, chosen from the single-emitter samples that yield sub-Poissonian emission in the same figure. Note that free-space dipole–dipole interactions are neglected in our calculations, which are valid approximations given the fact that EM local density of states at the gap region is completely governed by the cavity SP modes. In Figure 4a, both emitters are red-detuned with respect to the dipolar SP, while they are at the invisibility dip between the dipolar and quadrupolar modes in Figure 4b. Solid lines plot *g*^(2)^(0) for the two emitters, while dashed lines represent the associated single-emitter cases. In both panels, we consider QE frequencies slightly separated, ω_E2_ – ω_E1_ = 0.1 eV, which is of the order of the Drude damping, γ_m_. The correlation function for further QE–QE detunings is basically the superposition of the two single-emitter calculations. On the other hand, if ω_E_ is the same for both emitters, the plexciton emission is that of the single QE with a larger transition dipole moment. Figure 4 explores the intermediate regime: a significant enhancement of negative correlations is not apparent at low QE frequencies (a), but a strong reduction in *g*^(2)^(0) takes place for ω_E1_ and ω_E2_ at the invisibility dip. In particular, we observe a dip in the correlation function at laser frequencies between the two QE lines. In that minimum, *g*^(2)^(0) ∼ 0.3, while the corresponding single-emitter spectra do not present values below 0.7. Thus, we can conclude that indeed the interplay and interaction between various SP modes and QEs can be exploited to enhance the degree of antibunching in the photon emission by NPoM plexcitonic systems.

## Conclusion

We have presented a master equation description of the far-field photon emission by a plasmonic nanoparticle-on-mirror cavity strongly coupled to a single molecule or quantum emitter. We have employed our model, parameterized through classical electromagnetic calculations, to characterize the classical and quantum-optical properties of the light scattered by this hybrid system in a dark-field-like setup. First, we have found that the formation of plexcitons does not yield significant antibunching in the most explored sample configuration in which the molecular transition is at resonance with the dipolar cavity mode. Next, by varying the laser and emitter frequencies, we have explored the whole plasmonic spectrum of the nanostructure. This way, we have found that large negative photon correlations take place at three different emitter frequencies: below the dipolar plasmon, at the invisibility dip between this mode and the quadrupolar one, and at resonance with the plasmonic pseudomode. Finally, we have demonstrated that, under certain conditions, photon antibunching can be enhanced through the introduction of a second molecule in the nanocavity. We believe that our theoretical findings shed light into recent experiments and can serve as a guide for the design of devices for quantum light generation through the strong coupling of light and material states at the nanoscale.

## Supporting Information Available

Details on several aspects of the theory in the main text; the nanocavity description; the impact of metal nonlocality and retardation effects in the SP modes; characterization of their far-field radiative properties; the mode sensitivity to geometric facets in the gap region; the SP natural frequencies and dipole moments; the influence of the nonradiative decay and dephasing rates of the quantum emitter on the second-order correlation function; analysis of the mode contributions to the scattered intensity and correlations (PDF)
