# Supplementary material for: Plexcitonic Quantum Light Emission from Nanoparticle-on-Mirror Cavities
Source: Nano Lett. 2022 Mar 14;22(6):2365–73. doi: 10.1021/acs.nanolett.1c04872 (PMC8949753; doi:10.1021/acs.nanolett.1c04872)
Supplement: Supplementary file 1 — nl1c04872_si_001.pdf [file nl1c04872_si_001.pdf]

Supporting Information: Plexcitonic quantum light emission from nanoparticle-on-mirror cavities: Supplemental material

Rocío Sáez-Blázquez,[*,†,‡] Álvaro Cuartero-González,[†,¶] Johannes Feist,[†]
Francisco J. García-Vidal,[†,§] and Antonio I. Fernández-Domínguez[*,†]

†*Departamento de Física Teórica de la Materia Condensada and Condensed Matter Physics Center (IFIMAC), Universidad Autónoma de Madrid, 28049 Madrid, Spain*

‡*Vienna Center for Quantum Science and Technology, Atominstitut, TU Wien, 1040 Vienna, Austria*

¶*Mechanical Engineering Department, ICAI, Universidad Pontificia Comillas, 28015 Madrid, Spain*

§*Institute of High Performance Computing, Agency for Science, Technology, and Research (A*STAR), Singapore 138632, Singapore*

E-mail: rocio.saez.blazquez@tuwien.ac.at; a.fernandez-dominguez@uam.es

Abstract

This document provides supporting information to the letter *Plexcitonic quantum light emission from nanoparticle-on-mirror cavities.* It gives details on several aspects of the theory presented in the main text. First, we extend the nanocavity description in four ways. We analyze the impact of metal nonlocality and retardation effects in the surface plasmon (SP) modes, characterize their far-field radiative properties, explore the mode sensitivity to geometric facets in the gap region, and provide the SP

natural frequencies and dipole moments. Second, we investigate the influence of the nonradiative decay and dephasing rates of the quantum emitter (QE) on the second-order correlation function. Finally, we disentangle different SP modal contributions to the intensity and photon correlations by reducing the nanocavity spectrum in different forms.

Cavity description beyond the local, quasistatic limit

In recent years, much research attention has focused on the emergence of nonlocal effects in the permittivity of metallic nanostructures sustaining SP modes with effective volumes approaching the Fermi-Thomas wavelength[1–3]. This electromagnetic (EM) length scale, which sets the thickness of the induced charge density at the metal surface, is given by the ratio between the Drude plasma frequency ($\omega_{\mathrm{p}} = 8.91\,\mathrm{eV}$, see main text) and the Fermi velocity ($\beta \sim 10^6\,\mathrm{m/s}$), having $L_{\mathrm{F}} \approx \omega_{\mathrm{p}}/\beta \approx 0.1\,\mathrm{nm}$ for silver[1]. In the following, we investigate how these nonlocal effects affect the spectral density of our NPoM cavities with $\delta = 0.9$ nm, the ones featuring the largest EM localization. We employ a simplified nonlocal hydrodynamical model[2], whose implementation in standard EM simulation tools is straightforward, and that has been tested in geometries very similar to ours. Figure S1 shows $J(\omega)$ under the local approximation (solid blue line) and for three different Fermi velocities (dashed lines): 10^5 (red), 10^6 (orange) and 10^7 (yellow) m/s. We can observe that the nonlocal blueshift and reduction of SP maxima is much larger at high frequencies, approaching the pseudomode, than at the lowest, brightest SPs. In particular, at $\beta = 10^6\,\mathrm{m/s}$, the nonlocal reduction of $J(\omega)$ is of the order of a factor 1.4 at the dipolar plasmon and roughly 2 at the pseudomode. The impact of nonlocality is therefore smaller than shrinking the QE dipole moment by $\sqrt{2} \approx 1.4$. Moreover, recent reports indicate that this moderate impact of metal nonlocality in plasmon-emitter interactions is even smaller in systems, the so-called picocavities, that generate modal volumes below $1\,\mathrm{nm}^3$. In these structures, atomistic lightning-rod-like effects enhance light-matter interactions, a mechanism that is also neglected in our theory[4].

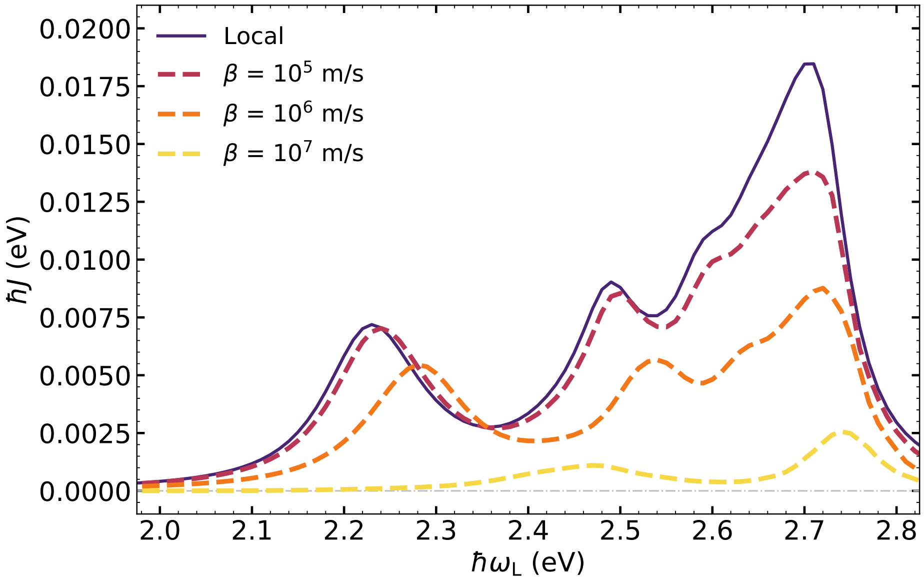

Figure S1: Impact of metal nonlocality on the spectral density of NPoM cavities with $D = 30\,\mathrm{nm}$ and $\delta = 0.9\,\mathrm{nm}$. The local spectrum (solid blue line) is compared against nonlocal predictions (red, orange and yellow dashed lines) for different Fermi velocities, β.

Figure S2(a) explores the validity of our analytical results for $J(\omega)$ at the gap center in the NPoM cavities of Figure 1 in the main text. Our analytical predictions (colored lines) are compared against both quasi-static (QS, grey dashed lines) and full electrodynamic (Full EM, black dotted lines) numerical simulations. The former are obtained through COMSOL simulations on NPoM cavities whose size is scaled down to remove retardation while keeping radiation effects. We can observe that their agreement with our analytical model, also constructed in the quasi-static limit, is almost perfect. The latter reveal the impact of retardation in the nanoparticles with $D = 30\,\mathrm{nm}$. We can observe that, as expected, this introduces a small redshift in the lowest frequency, most radiative SPs, but do not affect the high-order modes neither the pseudomode. Figure S2(b) shows the far-field radiation profile (radial Poynting vector versus azimuthal angle) for the three lowest SP modes in the cavity with $\delta = 0.9\,\mathrm{nm}$. These were obtained through full electrodynamic simulations evaluated at the three lowest-frequency maxima in $J(\omega)$: 2.23, 2.49 and 2.59 eV (see panel a). The results are normalized to the maximum of the Poynting vector for 2.23 eV. We can observe

that, although the induced surface charge distribution associated with these three SPs have different multipolar character (dipolar, quadrupolar and octupolar) the three modes present a dipolar-like emission pattern. As expected, they correspond to a vertically-oriented dipole on top of a metal substrate (mirror). This can be attributed to retardation effects, which provide an effective radiative dipole moment for the SP modes that present a non-dipolar character in the near-field. As expected, our calculations reveal that the radiated power decreases with increasing frequency and multipolar order.

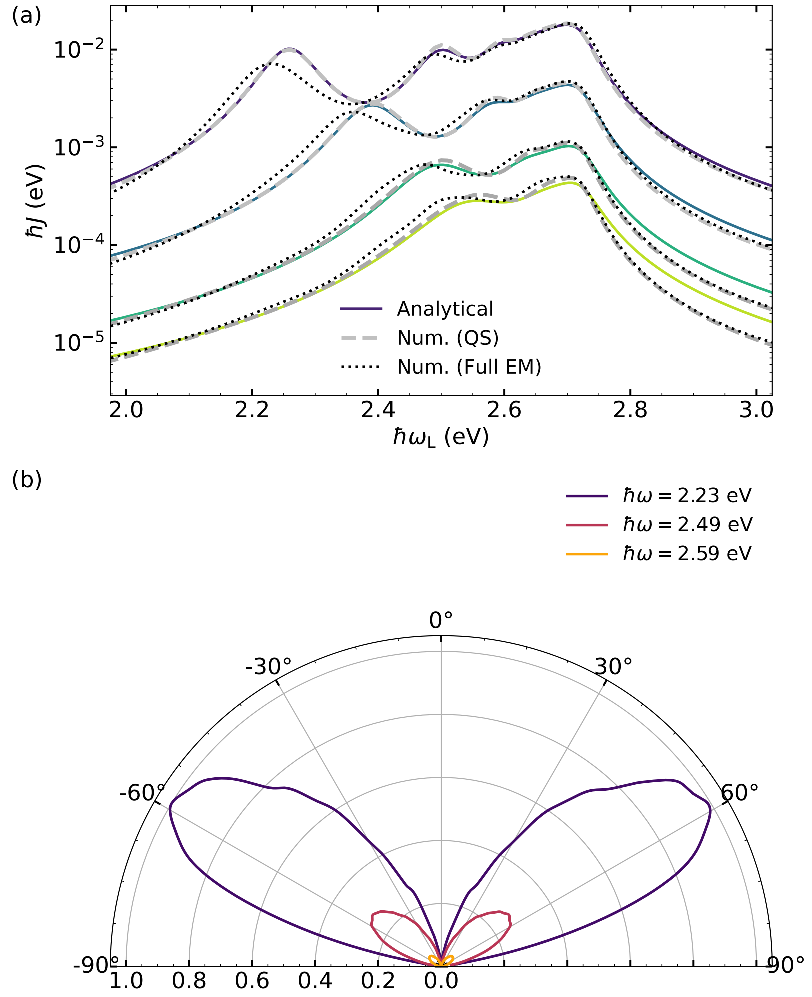

Figure S2: (a) Spectral density at the gap center of NPoM cavities with $D = 30\,\text{nm}$ and different δ. $J(\omega)$ obtained with our analytical model (colored solid lines) is compared against quasi-static (QS, dashed grey lines) and full electrodynamic (Full EM, dotted black lines) numerical simulations. (b) Numerical far-field emission profile (radial Poynting vector versus azimuthal angle) for the three lowest SP modes sustained by the cavity with $\delta = 0.9\,\text{nm}$.

Additionally, recent experimental reports on plasmonic nanogaps have shown the key role that nanoparticle facets can have in plasmon-exciton interactions[5,6]. In Figure S3, we plot the spectral densities associated with a nanoparticle-on-mirror NPoM cavity in the quasistatic limit with different shape truncations at the gap. The nanoparticle diameter ($D = 30\,$nm) and gap size ($\delta = 0.9\,$nm) are fixed in our simulations. The emitter is placed at the gap center and it is vertically oriented, ensuring its strongest coupling with the SP spectrum. The metal is described in a Drude-like permittivity for silver, like in the main text. Different sizes for the planar facet, w, have been considered, resulting in a negligible variation in the high-order SP mode contributions to the spectral density. The effect of the facet is only evident at low frequencies, yielding small redshifts in the dipolar mode frequency, as well as variations in the relative height of the different SP maxima in $J(\omega)$. Note that for large enough w, new peaks emerge in the spectral density due to the hybridization of NPoM modes and the standing waves of metal-insulator-metal SPs propagating along the facet[6].

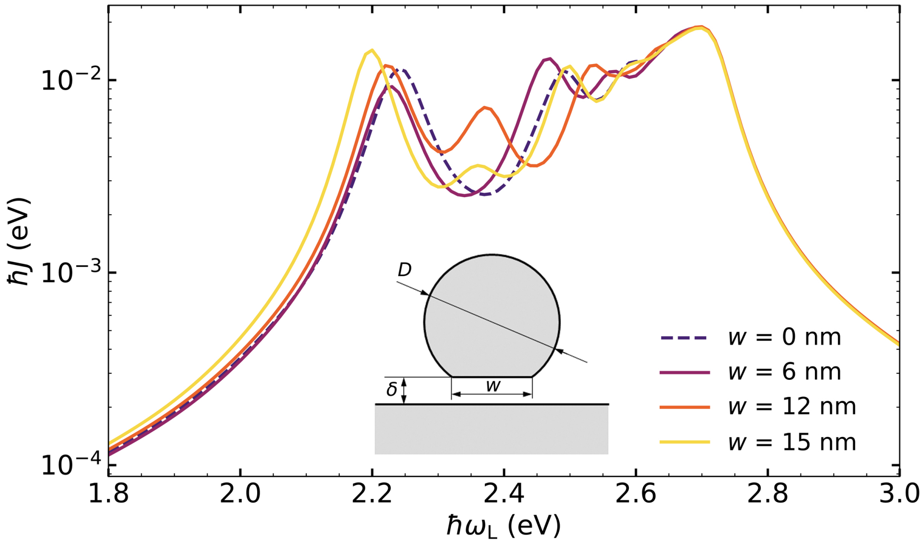

Figure S3: Spectral density $J(\omega)$ at the gap center of the NPoM flat-faceted cavity. The QE is vertically oriented, and the gap size and nanoparticle diameter are set to $\delta = 0.9\,$nm and $D = 30\,$nm. The size of the planar bottom facet w is varied (see inset).

Figure S4(a) shows the SP dipole moments extracted from our analytical model for NPoM cavities with different gap sizes[7,8]. Again, the parameters are the same as in Figure 1 of the

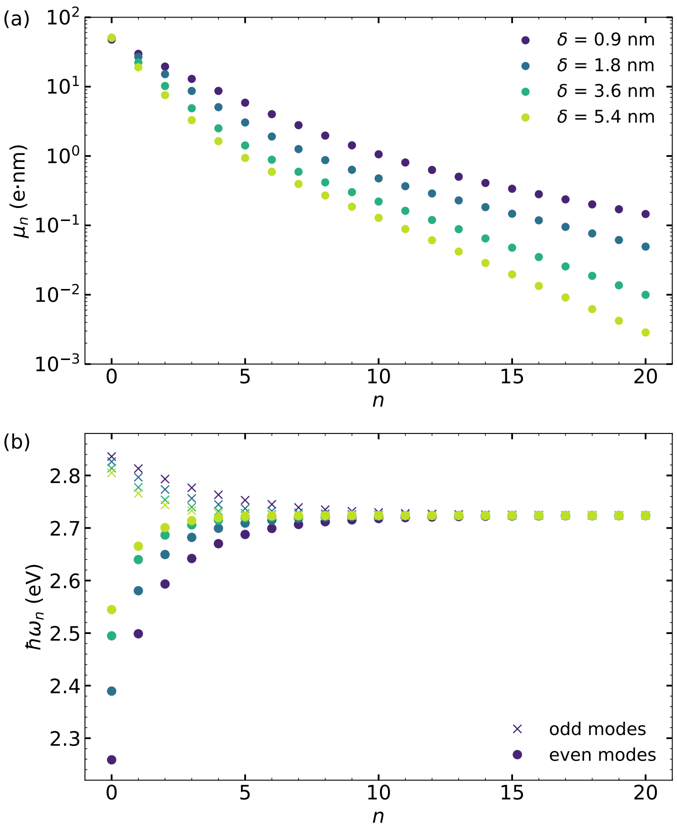

Figure S4: Modal dependence of the plasmonic modes for a NPoM cavity with $D = 30\,\text{nm}$ and different gap sizes δ. (a) Induced dipole moments μ_n as a function of the mode order n for even SP modes ($\alpha = \{1, n\}$). (b) Modal frequencies ω_n for each parity, with even ($\alpha = \{1, n\}$) and odd ($\alpha = \{-1, n\}$) modes associated to order n represented with different symbols.

main text. We can observe that the dipole moment for the even, dipolar SP ($\alpha = \{1, n\}$) is $\mu_1 \approx 50\,\text{nm}$ for all δ. The dipole moment for higher order SPs is significantly smaller for in all cases, but μ_n decays faster with n for larger δ. Note that only the SP modes with even parity have a finite dipole moment. There is a family of SP modes contributing to $J(\omega)$ which present odd parity and are dark, presenting a vanishing dipole moment. Figure S4(b) plots the natural frequencies for the SP modes of the structures in panel (a). We can observe two different branches in ω_n, which correspond to SP modes with even (low-frequency, dots) and odd (high-frequency, thin crosses) parity. Both branches merge at large n at the pseudomode frequency, which emerges at the asymptotic surface plasmon frequency of the flat metal-vacuum interface (see main text).

Effect of QE nonradiative decay and dephasing

The precise values of the second-order correlation function strongly depend on the parameters of the system. We have shown in the main text, both via de gap size and the emitter position, that the NPoM-QE samples that provide larger light-matter interaction also produce stronger photon correlations. Concerning the spectral characteristics of the QE, it has been shown that large nonradiative losses are beneficial for interference-induced antibunching. On the contrary, they are detrimental to reach low values of $g^{(2)}(0)$ via photon blockade[9]. In order to gain a better understanding of the effect of emitter nonradiative decay and dephasing in the main features of the photon statistics phenomenology in Figure 3 of the main text, we vary next both γ_{nrad} and γ_{deph} and analyze how the $g^{(2)}(0)$ changes in the most relevant regions of antibunching.

Figure S5 renders the second-order correlation function versus laser frequency for the two QE positions analyzed in the main text, namely, at the gap center ($z_{\mathrm{E}} = 0.5\delta$, panel a) and vertically displaced ($z_{\mathrm{E}} = 0.85\delta$, panel b). We consider two QE frequencies, which lie in two different configurations of sub-Poissonian emission: below the dipolar SP mode ($\omega_{\mathrm{E}} = 2.00\,\mathrm{eV}$) in the left panels, and between the dipolar and the quadrupolar SPs ($\omega_{\mathrm{E}} = 2.40\,\mathrm{eV}$) in the right panels. Colors represent the values of the dephasing rate ($\gamma_{\mathrm{deph}} = 1\,\mathrm{meV}$ in dark blue, and $10\,\mathrm{meV}$ in light blue) and line styles, the nonradiative rates ($\gamma_{\mathrm{nrad}} = 0\,\mathrm{meV}$ in solid lines, and $10\,\mathrm{meV}$ in dashes lines). It is evident that molecular dephasing always diminishes photon correlations (indeed, both the maximum and minimum of $g^{(2)}(0)$ are reduced). Nevertheless, nonradiative decay increases negative photon correlations at the invisibility dip (right panels) and affects them in different ways at $\omega_{\mathrm{E}} < \omega_{\mathrm{dip}}$ (left panels). We can observe that a lower (larger) photon antibunching is attained in the latter case for laser frequencies red-detuned (blue-detuned) from the QE frequency. As previously discussed, this distinctive behaviour is related with the different origin of the sub-Poissonian emission. As a consequence, QEs with lower quantum yield may be more convenient for the generation of nonclassical light in NPoM cavities depending on the plexcitonic configuration.

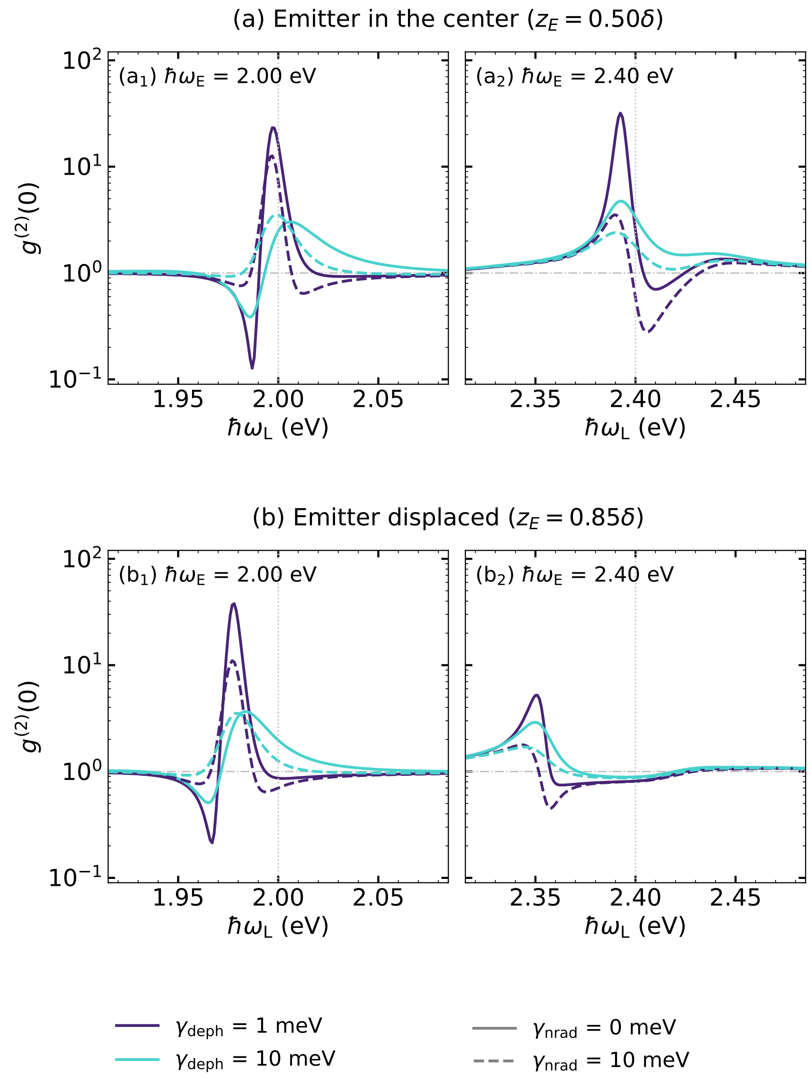

Figure S5: Second-order correlation function $g^{(2)}(0)$ versus laser frequency ω_L for QE at the gap center (a) and displaced vertically (b). Two emitter frequencies are considered, lying in different antibunching regions: red detuned with respect to the dipolar mode, $\omega_E = 2.00\,\mathrm{eV}$ (left), and within the invisibility dip, $\omega_E = 2.40\,\mathrm{eV}$ (right). In all panels, line colours code the QE dephasing, while line styles, the nonradiative decay. Vertical dotted lines indicate the position of the QE frequency (all SP frequencies are outside the spectral window shown).

Analysis of the mode contributions to the scattered intensity and correlations

Finally, we compare the intensity and the second-order correlation function obtained from the full plasmonic spectrum of the nanocavity and those resulting from its restriction to a

few SP modes. The dashed line in Figure S6 represents the spectral density of a NPoM cavity with $D = 30\,\mathrm{nm}$ and gap size $\delta = 0.9\,\mathrm{nm}$, as the one considered in the main text. In contrast, continuous colored lines plot the spectral densities obtained when only the first 1, 2, 4, and 6 SP modes are included in $J(\omega)$. We can observe that, as expected, the simplified descriptions of the nanocavity fail at higher frequencies as the number of modes increases.

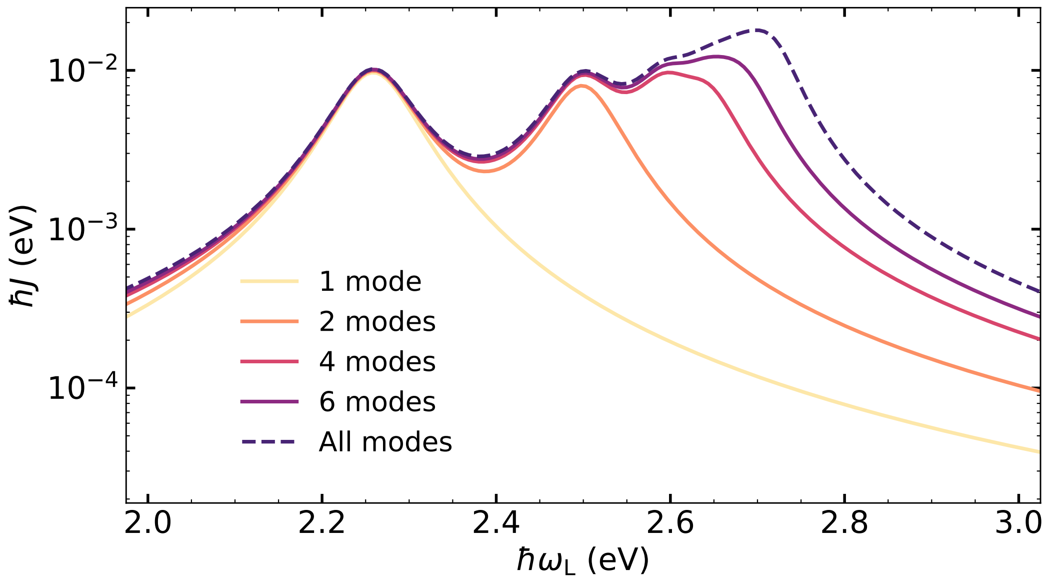

Figure S6: Spectral densities at the gap center for $(D, \delta) = (30, 0.9)\,\mathrm{nm}$ resulting from considering only the first 1, 2, 4, and 6 modes of the nanocavity (continuous lines), together with the complete plasmonic spectrum (dashed line).

The characteristics of the nanocavity emission are not reproduced when the reduced spectral densities in Figure S6 are used in the description of the nanostructure. In Figure S7 and Figure S8, we plot the scattering intensity I_{scat} (top panels) and the second-order correlation function $g^{(2)}(0)$ (bottom panels) obtained for the complete plasmonic spectrum (dashed line), and for its simplification into a few modes only (continuous lines). We consider again the two QE positions inside the cavity: gap center (a) and vertically displaced (b). When the emitter frequency lies below the the dipolar mode ($\omega_{\mathrm{E}} = 2.00\,\mathrm{eV}$, Figure S7), a few modes are sufficient to reproduce the effect of the complete spectrum if the emitter is at the center of the gap (panels a). Nevertheless, when the emitter is displaced with respect to this cen-

tral position, shifts in both the intensity maxima and the antibunching minima are observed (panels b). This is produced by the increased coupling to the high-frequency pseudomode in this configuration[7]. Besides, a region of moderate antibunching for frequencies above the emitter natural frequency develops only when the whole spectrum is considered.

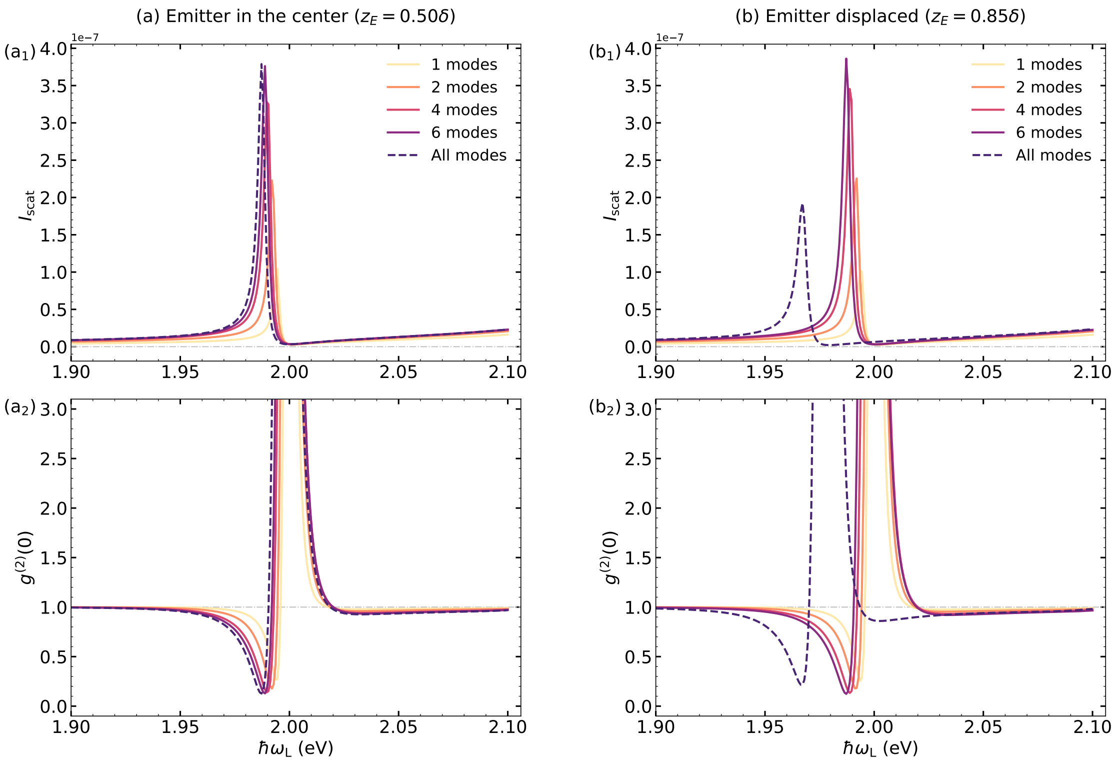

Figure S7: Scattering intensity $I_{\rm scat}$ (top panels) and second-order correlation function $g^{(2)}(0)$ (bottom panels) as a function of the laser frequency $\omega_{\rm L}$ for a QE frequency $\omega_{\rm E} = 2.00\,$eV placed in the center of the gap (a) or displaced (b).

When the emitter frequency lies between the dipolar and the quadrupolar SP modes ($\omega_{\rm E} = 2.40\,$eV, Figure S8), the picture varies. Even when the emitter is at the gap center (panels a), the second-order correlation function is not reproduced unless a considerable number of modes are included in $J(\omega)$. While a region of antibunching is observed around $\omega_{\rm L} = 2.41\,$eV for the complete plasmonic spectrum, the correlation function does not present values below 1 when the it is reduced to the first six modes. When the emitter is displaced with respect to the cavity center (panels b), the frequency window with antibunched emission

broadens. Interferences between the different higher-order modes lead to diverse shifts of the minima displayed in the intensity and second-order correlation functions. All these results highlight that accounting for the full complexity of the nanocavity SP spectrum is crucial in order to describe accurately its performance for nonclassical light generation.

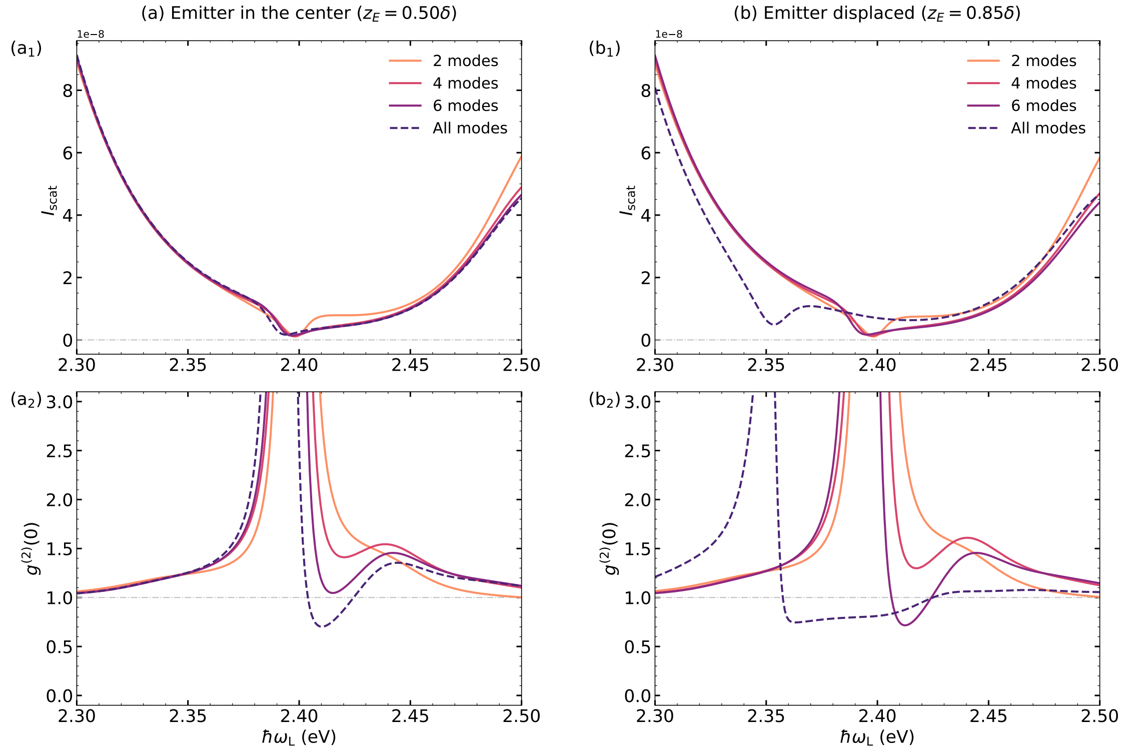

Figure S8: Scattering intensity I_{scat} (top panels) and second-order correlation function $g^{(2)}(0)$ (bottom panels) as a function of the laser frequency ω_{L} for a QE of frequency $\omega_{\text{E}} = 2.40\,\text{eV}$ placed in the center of the gap (a) or displaced (b).

References

(1) Ciraci, C.; Hill, R. T.; Mock, J. J.; Urzhumov, Y.; Fernández-Domínguez, A. I.; Maier, S. A.; Pendry, J. B.; Chilkoti, A.; Smith, D. R. Probing the Ultimate Limits of Plasmonic Enhancement. *Science* **2012**, *337*, 1072–1074.

(2) Luo, Y.; Fernandez-Dominguez, A. I.; Wiener, A.; Maier, S. A.; Pendry, J. B. Surface Plasmons and Nonlocality: A Simple Model. *Phys. Rev. Lett.* **2013**, *111*, 093901.

(3) Mortensen, N. A.; Raza, S.; Wubs, M.; Søndergaard, T.; Bozhevolnyi, S. I. A generalized non-local optical response theory for plasmonic nanostructures. *Nature Communications* **2014**, *5*, 1–7.

(4) Benz, F.; Schmidt, M. K.; Dreismann, A.; Chikkaraddy, R.; Zhang, Y.; Demetriadou, A.; Carnegie, C.; Ohadi, H.; de Nijs, B.; Esteban, R.; Aizpurua, J.; Baumberg, J. J. Single-molecule optomechanics in “picocavities”. *Science* **2016**, *354*, 726–729.

(5) Ojambati, O. S.; Chikkaraddy, R.; Deacon, W. D.; Horton, M.; Kos, D.; Turek, V. A.; Keyser, U. F.; Baumberg, J. J. Quantum electrodynamics at room temperature coupling a single vibrating molecule with a plasmonic nanocavity. *Nature Communications* **2019**, *10*, 1–7.

(6) Baumberg, J. J.; Aizpurua, J.; Mikkelsen, M. H.; Smith, D. R. Extreme nanophotonics from ultrathin metallic gaps. *Nature Materials* **2019**, *18*, 668–678.

(7) Cuartero-González, A.; Fernández-Domínguez, A. I. Light-Forbidden Transitions in Plasmon-Emitter Interactions beyond the Weak Coupling Regime. *ACS Photonics* **2018**, *5*, 3415–3420.

(8) Cuartero-González, A.; Fernández-Domínguez, A. I. Dipolar and quadrupolar excitons coupled to a nanoparticle-on-mirror cavity. *Phys. Rev. B* **2020**, *101*, 035403.

(9) Sáez-Blázquez, R.; Feist, J.; García-Vidal, F. J.; Fernández-Domínguez, A. I. Photon statistics in collective strong coupling: Nanocavities and microcavities. *Phys. Rev. A* **2018**, *98*, 013839.
